# The Need for Expanding Pulmonary Rehabilitation Services

**DOI:** 10.3390/life11111236

**Published:** 2021-11-15

**Authors:** Aroub Lahham, Anne E. Holland

**Affiliations:** 1 Department of Immunology and Pathology, Monash University, Melbourne 3800, Australia; Anne.Holland@monash.edu; 2 Institute for Breathing and Sleep, Melbourne 3084, Australia; 3 Department of Physiotherapy, Alfred Health, Melbourne 3004, Australia

**Keywords:** pulmonary rehabilitation, health care services, chronic respiratory disease, health service design, COVID-19, integrated care

## Abstract

Pulmonary rehabilitation is a strongly recommended and effective treatment for people with chronic lung disease. However, access to pulmonary rehabilitation is poor. Globally, pulmonary rehabilitation is accessed by less than 3% of people with chronic lung disease. Barriers to referral, uptake and completion of pulmonary rehabilitation are well documented and linked with organizational, practitioner and patient-related factors. Enhancing the knowledge of health care professionals, family carers, and people with chronic lung disease about the program and its benefits produces modest increases in referral and uptake rates, but evidence of the sustainability of such approaches is limited. Additionally, initiatives focusing on addressing organizational barriers to access, such as expanding services and implementing alternative models to the conventional center-based setting, are not yet widely used in clinical practice. The COVID-19 pandemic has highlighted the urgent need for health care systems to deliver pulmonary rehabilitation programs remotely, safely, and efficiently. This paper will discuss the pressing need to address the issue of the low accessibility of pulmonary rehabilitation. It will also highlight the distinctive challenges to pulmonary rehabilitation delivery in rural and remote regions, as well as low-income countries.

## 1. Definition and Benefits of Pulmonary Rehabilitation

Pulmonary rehabilitation is an important treatment for people with chronic lung disease [1]. The official American Thoracic Society (ATS)/European Respiratory society (ERS) statement defined pulmonary rehabilitation as ‘a comprehensive intervention based on a thorough patient assessment followed by patient-tailored therapies that include, but are not limited to exercise training, education, and behavior change, designed to improve the physical and psychological condition of people with chronic respiratory disease and to promote the long-term adherence to health-enhancing behaviors’ [2]. The recent official ATS Workshop Report on defining pulmonary rehabilitation further identified 13 essential components of pulmonary rehabilitation across domains of patient assessment (exercise capacity, quality of life, dyspnea, nutrition and occupational status), program content (endurance and resistance training), method of delivery (exercise programs that are individually prescribed and progressed by experienced staff) and quality assurance (adequate training for health professionals) [3].

In people with chronic obstructive pulmonary disease (COPD), strong evidence confirms that pulmonary rehabilitation optimizes muscle function, improves exercise capacity, reduces dyspnea and enhances health-related quality of life [4]. Further evidence demonstrates that pulmonary rehabilitation offered early after hospital discharge from an exacerbation of COPD can reduce the likelihood of readmission by 56% [5]. In people with idiopathic pulmonary disease (IPF), a recent Cochrane review (updated 2021) found pulmonary rehabilitation to be safe and effective, with clinically meaningful benefits for patients [6]. Indeed, individuals with IPF who had received pulmonary rehabilitation reported physical, psychological and social gains [7]. A growing body of evidence also reports beneficial effects of pulmonary rehabilitation in people with bronchiectasis and pulmonary hypertension [8,9]. The impact of pulmonary rehabilitation on people with chronic lung disease is summarized in Table 1. Despite these important benefits, pulmonary rehabilitation is universally underutilized, and referral, uptake and completion rates are alarmingly low. This paper will highlight the urgent need to expand pulmonary rehabilitation services, and the potential role for tele-medicine to enhance pulmonary rehabilitation outcomes.

## 2. Barriers to Accessing Pulmonary Rehabilitation

**Figure 1:**
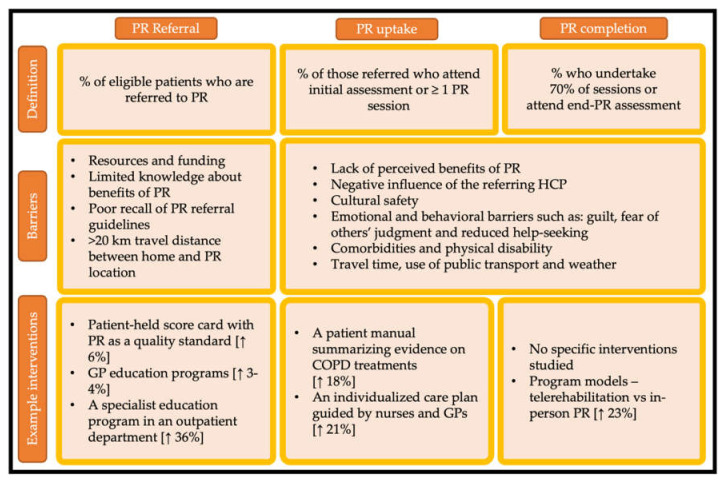
Common barriers and studied interventions of pulmonary rehabilitation access across the domains of referral, uptake and completion. PR: pulmonary rehabilitation; HCP: health care professionals; GPs: general practitioners. Data are from references [13,14,15,16,17,18,19].

Decades of research has demonstrated consistent barriers to access to pulmonary rehabilitation. The official ATS/ERS statement of 2015 highlighted important barriers to pulmonary rehabilitation including insufficient funding, limited resources and lack of knowledge and skill of healthcare professionals and patients regarding the benefits of pulmonary rehabilitation [11]. In 2017, a systematic review using the theoretical domains framework studied barriers to referral, uptake and participation in 48 scientific reports [12]. Overall, 70% of included articles identified environmental barriers, 38% reported barriers related to lack of knowledge about pulmonary rehabilitation and 31% reported barriers related to beliefs about consequences [12]. Common barriers to pulmonary rehabilitation across the domains of referral, uptake and completion are shown in Figure 1, along with examples of interventions that aim to address the critical determinants of pulmonary rehabilitation participation.

## 3. Referral to Pulmonary Rehabilitation

While there has been a modest improvement over the last decade, current rates of referrals to pulmonary rehabilitation in COPD remain suboptimal worldwide. In 2018, a scoping review of 10 developed countries found referral rates of 35% of eligible patients or lower in over 90% of included studies [20]. A more recent United Kingdom (UK) audit reported that in primary care, only 16% of people with COPD were referred to pulmonary rehabilitation [21]. Referral rates appear to be similar in developing countries. A recent survey of health care professionals and people with COPD in Uganda reported that referral rates to pulmonary rehabilitation were only 23%, despite high interest in the program from 92% of patients surveyed [22]. Referral rates appear to be lowest post-hospitalization due to exacerbation of COPD based on a recent audit, with only 1.9% of patients receiving pulmonary rehabilitation within 6 months post-hospitalization and 2.7% within 12 months [23,24]. For people with IPF, referral rates are poorly reported worldwide. A recent United States (US) analysis from an American IPF registry showed low rates of referrals of 727 individuals with IPF, with only 19% referred [25].

In a recent editorial in *JAMA*, Rochester et al. highlighted that one of the key barriers to pulmonary rehabilitation referral was the poor recognition of its benefits by health care organizations [26]. This is partially represented by the limited resources and funding that are dedicated to pulmonary rehabilitation services. In 2015 worldwide, pulmonary rehabilitation was available to 2.7% of people with COPD [27]. A US study reported that 59% of US counties do not have a hospital outpatient program located in its tertiary hospitals [28]. Additionally, only 70 Australian outpatient programs were available in the regional and rural areas that are home to 30% of the Australian population [29]. A Canadian survey reported that in 2015, the services of Canadian programs offered access to 0.4% of patients with COPD [30].

The two most common barriers to referral are related to the lack of healthcare professionals’ knowledge: knowledge about the construct of pulmonary rehabilitation and knowledge about its benefits [11]. In the US, 61% of 154 respiratory clinicians had no knowledge about any health care recommendations for pulmonary rehabilitation referral [31]. A German cross-sectional survey of 590 pulmonary specialists reported that 62% perceived the program to be suitable for patients with moderate COPD, while 31% regarded it necessary in severe COPD [32]. In the UK, general practice patients were more likely to be referred if they were depressed, had a recent exacerbation of COPD or were ex-smokers [21]. In another survey-based study in Sri Lanka, 83% health care professionals were unsure of what made their patients eligible for the program [33]. In Uganda, 77% of health care professionals did not have enough information about the program while 47% identified lack of time to complete the referral process [22]. Referral rates in people after exacerbation of COPD were lower in staff who did not perceive confidence in pulmonary rehabilitation prescription and had poor recall of care recommendations for referral [24]. Patient travel time was also regarded as a barrier to rehabilitation referral, as health care professionals in New Zealand reported that they were less likely to refer patients to an on-site rehabilitation center that is located more than 20 km from their patient’s residential address [34].

## 4. Uptake and Completion of Pulmonary Rehabilitation

Pulmonary rehabilitation uptake, which has been defined as the percentage of referred patients who attend the program for their initial assessment or attend at least one session [3], is also inadequate. A UK audit in 2013–2014 estimated that of 68,000 people with COPD who were referred to pulmonary rehabilitation, 69% received their initial assessment and first session [35]. Another audit from New Zealand reported similar percentages, with 80% of referred patients attending at least one session [36]. Uptake in patients post-hospital discharge due to exacerbation of COPD is even lower. A UK audit reported that, of 90 pulmonary rehabilitation referrals arranged after discharge from hospital due to exacerbation of COPD, 68 patients attended their initial rehabilitation assessment and only 60 commenced the program [23].

Completion of pulmonary rehabilitation has been defined as the percentage of attendees who underwent 70% of sessions or those who completed their end-rehabilitation assessment [3]. It is reported that 10–32% of people who start do not complete the program [37,38]. A national New Zealand survey conducted in the low-population density of southern regions reported completion rates of 3% of people with severe COPD who attended pulmonary rehabilitation [34]. Completion rates appeared more promising in the UK as a recent audit reported 67% of individuals who attended undertook end-rehabilitation assessment [39]. A predictor analysis reported that people with low socioeconomic status, more severe disease and more breathlessness had lower odds of pulmonary rehabilitation completion [21].

Lack of perceived benefits of rehabilitation is a strong barrier to program uptake [40]. One qualitative review reported that patients perceived negative associations with the name of the treatment, “*Rehab sounds like…when we used to take people to rehab centers sort of thing, old people, something like that, which would turn me off actually*” [41]. Perceptions about pulmonary rehabilitation were also influenced by cultural and educational backgrounds. The impact of cultural background, especially that of indigenous populations, is often overlooked by health care providers’ lack of understanding of ‘cultural safety’ [42]. Cultural safety is defined as “*a focus for the delivery of quality care through changes in thinking about power relationships and patients ‘rights’*” [42]. In the context of pulmonary rehabilitation, health care professionals should be skilled in building culturally safe interactions with rehabilitation attendees from diverse cultural backgrounds. This could be achieved through adequate levels of critical consciousness and empathy, which facilitate health equity amongst attendees [43]. Another barrier to uptake is the negative influence that could be given to the program by the referring physician, “*this may or may not help you!*” [44].

In another qualitative study in people who declined pulmonary rehabilitation post-exacerbation of COPD, guilt, fear of others’ judgment and reduced help-seeking appeared to be a strong barrier to uptake, “*I think sometimes the doctors can be very abrupt with you, ... I don’t know whether it’s my imagination … perhaps my guilt thinking oh perhaps they haven’t got much patience with me because it’s self-inflicted, I don’t know.*” [45]. People with COPD often fear that the intensity of the program will exceed their physical capabilities [38]. Additionally, unsuccessful previous experiences with management of COPD may negatively influence uptake of current referrals [46].

Other important barriers to uptake of pulmonary rehabilitation occur at the organizational level. Environmental factors such as travel time, use of public transport, and weather conditions are well-known barriers of rehabilitation uptake [30,40,47,48]. Longer travel time has been reported as a predictor for non-adherence to rehabilitation in the UK [49]. In Australia, a qualitative study explored the complex contributions of travel time as a barrier [48]. One reason was the inaccessibility of public transport or a car, “*I just can’t make it because I have no car and I have to walk all the way down to X Rd; that takes me about half an hour.*” (p3) [48]. Another reason was the cost associated with travel, which is not always covered by outpatient services, and the impact of physical comorbidities on functional and community mobility [48].

## 5. Challenges of Pulmonary Rehabilitation for Vulnerable Populations

### 5.1. Pulmonary Rehabilitation in Remote Regions

While access to rehabilitation services is poor worldwide, living in remote regions or rural areas acts as an added disadvantage for people with chronic lung disease. A US survey indicated that pulmonary rehabilitation centers are more established in metropolitan counties, while 73% of rural counties do not have an outpatient program [28]. In Australia, health service delivery variations were observed in the management of people post-exacerbation of COPD in both rural and urban locations [50]. Interventions to address uptake of rehabilitation in rural areas are scarce [51,52]. One Australian study tested the impact of an educational program followed by an update workshop on pulmonary rehabilitation in rural areas and in remote regions [52]. Following this program, three-locally run pulmonary rehabilitation services that met the Australian practice guidelines were established [52]. However, the sustainability of these programs and its implementation in practice are not assured, given that the remote services rely solely on internal funding or in-kind support [52]. A recent review of pulmonary rehabilitation services in rural areas in Australia and New Zealand indicated the limited literature on cost-effectiveness of care models in these areas [53].

### 5.2. Pulmonary Rehabilitation Centers in Developing Countries

A recent systematic review of 112 publications in 78 developing and underdeveloped countries found that pulmonary rehabilitation is established in only 17 of the included countries [54]. A qualitative study of 11 people with COPD and 19 family caregivers explored the barriers to access of pulmonary rehabilitation in Iran. Two distinctive major themes were highlighted concerning the inaccessibility of the service and the inadequate insurance available to cover the program [55]. Another review on the efficiency of rehabilitation services in low-income countries reported that implementation was likely to be successful if it was delivered with minimal resources, as funding is very limited [56]. Initiatives to improve access to pulmonary rehabilitation in developing countries with cultural adaptations are currently underway through the Global RECHARGE program, comprising collaborations between the National Institute for Health Research in UK and partners in India, Sri Lanka, Kyrgyzstan and Uganda [57]. To date, protocols of RCTs aiming to study the impact of pulmonary rehabilitation in Uganda, [58] and Sri Lanka [59] are being issued.

## 6. Interventions Aiming to Improve Referral Rates

Enhancing the knowledge of health care professionals on pulmonary rehabilitation has been prompted by the insights of health care professionals themselves, “*If we know what happens (in pulmonary rehabilitation) then we can sell it better (practice nurse)*” [60]. A systematic review of 10 studies that tested interventions to improve referral rates described significant increases in referral rates using three different interventions [61]: a patient-held score card containing pulmonary rehabilitation as an a important standard of quality care (a quasi-experimental study, 6% increase in referral rates) [13], general practice educational programs in primary care (longitudinal studies, 3–4% increase in referral rates) [14,15] and a clinician educational program in a hospital outpatient department (longitudinal cohort study, 36% increase in referral rates) [16]. Unfortunately, these educational programs have had a modest impact on referral rates. The review further highlighted that none of the referral interventions were targeted to optimize the accessibility of rehabilitation programs on a system-level, but rather focused on enhancing the knowledge of patients attending the service and upskilling health care professionals involved in COPD care [61].

One retrospective analysis reported that a Canadian hospital that includes a Living Well with COPD (LWW COPD) program had an 85% referral rate to pulmonary rehabilitation compared to hospitals without LWW COPD (12% referral rate) [62]. Additionally, 100% of the patients in the LWW COPD hospital received advice about pulmonary rehabilitation from their respiratory specialists [62]. One of the key goals of the LWWCOPD program is to facilitate the different settings of pulmonary rehabilitation practice, including ‘satellite’ modes of rehabilitation delivery in partnership with primary care institutions [63]. Optimizing the accessibility of rehabilitation services through satellite centers linked to an experienced tertiary site could be a promising approach. Further research should be directed to understand the relationship between service expansion and referral rates.

## 7. Interventions Aiming to Improve Uptake and Completion

Two systematic reviews appraised interventions to optimize uptake of pulmonary rehabilitation [61,64]. One systematic review [61] described two programs that produced statistically significant increases in uptake: a patient manual summarizing evidence for COPD treatments (a non-randomized controlled before and after trial, 18% increase in uptake rates) [17] and an individualized care plan supported by a partnership between nurses and general practitioners (a clustered RCT, 21% increase in uptake) [18]. Similar to interventions aiming to optimize referral, environmental barriers are often overlooked in the current literature. A second systematic review [64] included one controlled trial (but not randomized nor blinded) that added a computer tablet to support exercise training in pulmonary rehabilitation versus rehabilitation alone [65]. Inclusion of the tablet aimed to increase patient motivation for home training and direct reporting of exercise to health care professionals online [65]. Completion rates were higher in the intervention group (17.4% vs. 8.7% in the control group) but differences did not reach statistical significance [65]. The review also acknowledged the need for more interventions aiming to address uptake, including environmental modification and service expansion [64]. Another highlighted gap is that studies were conducted in high-income countries and there were no interventions in low-to-middle income countries, where over 90% of global deaths from COPD occur [66].

## 8. Access to Telerehabilitation and Home-Based Rates

From the start of the twenty-first century, tele-medicine has been reported to have many benefits, especially in providing care in a non-emergency setting [67]. Benefits such as improving access to care and reducing the use of health care resources are well-documented in clinical practice [68]. Indeed, a systematic review of qualitative analyses of patient-perceived benefits of tele-medicine included, reduced travel time, effective communication, ease of access, and low cost [69]. In 2015, an ATS/ERS statement on pulmonary rehabilitation highlighted the urgent need to find novel programs as an alternative to the traditional outpatient service and stated that this was an essential approach to addressing the underutilization of the current model [11]. Over recent decades, deliberate efforts were devoted to evaluate the effects of alternative models in people with COPD [70]. Indeed, a number of robust randomized controlled trials have shown similar benefits with home-based pulmonary rehabilitation and tele-rehabilitation to the center-based model [71,72,73,74]. Furthermore, two recent meta-analyses showed no significant difference in exercise capacity and quality of life gains between the two models [75,76]. Additionally, a recently published Cochrane review of telerehabilitation in chronic lung disease reported higher completion rates of telerehabilitation versus in-person or traditional pulmonary rehabilitation (93% versus 70%, respectively) [19].

These novel models of rehabilitation are not broadly adopted in usual care worldwide, although the COVID-19 pandemic has accelerated uptake. Small scale implementation studies carry promising results. In Australia, the HomeBase model from the HomeBase trial [72] was rolled out into clinical practice [77]. Over the course of one year, 36% of those referred to pulmonary rehabilitation chose to undertake home-based rehabilitation, 71% attended their initial assessment and 75% of those completed the program [77]. Roll-out participants indicated that they would not have attended pulmonary rehabilitation if only center-based care was offered [77]. Successful implementation on a broader scale requires consideration of policy and reimbursement drivers, as well as robust quality assurance processes [3].

## 9. Pulmonary Rehabilitation during COVID-19 Pandemic

People with chronic lung disease are amongst the most vulnerable groups to developing severe complications with COVID-19 [78]. The World Health Organization advised patients to stay at home and avoid social contact to the maximum extent possible [79]. This increased susceptibility to physical deconditioning and exercise avoidance. The pandemic forced a rapid pivot to remote services, which now highlights the urgent need for robust models with strong quality assurance [80]. A recent systematic review on the use of tele-medicine during COVID-19 reported that tele-medicine improves the provision of quality health services while minimizing the risk of COVID-19 transmission [81]. As a preliminary response to prompt clinical practice implementations of remotely delivered pulmonary rehabilitation, several health care platforms included resources for tele-rehabilitation and home-based rehabilitation, including the homebased rehabilitation website: https://homebaserehab.net/ (accessed on 13 October 2021). Additional remotely delivered pulmonary rehabilitation recourses include the Canadian Thoracic Society pulmonary rehabilitation-endorsed program and guidance on virtual models of pulmonary rehabilitation from the Agency for Clinical Innovation in New South Wales, Australia [82,83].

## 10. Conclusions

Access to pulmonary rehabilitation is suboptimal worldwide. Whilst barriers to referral, uptake and completion are well described, there have been few interventions that successfully modify these factors. Improving access to pulmonary rehabilitation through the use of telerehabilitation and home-based models shows promise but requires policy drivers for widespread implementation. The COVID-19 pandemic has highlighted the need to optimize the remote delivery of pulmonary rehabilitation services, with strong quality assurance to ensure optimal patient outcomes.

## Figures and Tables

**Table 1:** Benefits of pulmonary rehabilitation on people with chronic lung disease reported in systematic reviews.

Respiratory Conditions	Maximal Exercise Capacity	Functional Exercise Capacity	Quality of Life	Dyspnea	Re-Admission Due to Exacerbation
Chronic obstructive pulmonary disease	Peak workload (MD 6.77 Watts, 95% CI 1.89 to 11.65; 16 studies) [4]	Six-minute walk distance (MD 43.93 m, 95% CI 32.64 to 55.21; 38 studies) [4]	SGRQ total (MD −6.89, 95% CI −9.26 to −4.52; 19 studies) [4]	Modified Borg Scale (MD −0.62 points, 95% CI −1.10 to −0.14; 12 studies) [10]	Re-admissions (OR 0.44, 95% CI 0.21 to 0.91; 8 studies) [5]
Idiopathic pulmonary fibrosis	Peak workload (MD 9.04 Watts, 95% CI 6.07 to 12.0; 4 studies) [6]	Six-minute walk distance (MD 40.07 metres, 95% CI 32.70 to 47.44; 13 studies) [6]	SGRQ total (MD −9.29, 95%CI −11.06 to −7.52; 11 studies) [6]	mMRC: MD) −0.36, 95% CI −0.58 to −0.14; 7 studies) [6]	NR
Pulmonary hypertension	Peak workload (MD 16.4 watts, 95% CI 10.9 to 22.0; 4 studies) [9]	Six-minute walk distance (MD 60.12 m, 95% CI 30.17 to 90.07; 5 studies) [9]	SF-36 physical (MD 4.63, 95% CI 0.80 to 8.47; 2 studies) SF-36 mental (MD 4.17, 95% CI 0.01 to 8.34; 2 studies) [9]	NR	NR
Non-cystic fibrosis bronchiectasis	NR	Incremental shuttle walk distance (MD 67 m, 95% CI 52 to 82; 3 studies) [8]	SGRQ total (MD −4.65; 95% CI, −6.7 to −2.6, 2 studies) [8]	NR	NR

MD: Mean difference between pulmonary rehabilitation and usual care; SGRQ: St George respiratory questionnaire; CI: Confidence interval; OR: Odds ratio; mMRC: modified medical research council; NR: Not reported.

## Data Availability

Not applicable.

## References

[1] (2021). Global Initiative for Chronic Obstructive Lung Disease. The Global Strategy for Diagnosis, Management and Prevention of COPD. https://goldcopd.org/2021-gold-reports/.

[2] Spruit M.A., Singh S.J., Garvey C., ZuWallack R., Nici L., Rochester C., Hill K., Holland A.E., Lareau S.C., Man W.D. (2013). An official American Thoracic Society/European Respiratory Society statement: Key concepts and advances in pulmonary rehabilitation. Am. J. Respir. Crit. Care Med..

[3] Holland A.E., Cox N.S., Houchen-Wolloff L., Rochester C.L., Garvey C., ZuWallack R., Nici L., Limberg T., Lareau S.C., Yawn B.P. (2021). Defining Modern Pulmonary Rehabilitation. An Official American Thoracic Society Workshop Report. Ann. Am. Thorac. Soc..

[4] McCarthy B., Casey D., Devane D., Murphy K., Murphy E., Lacasse Y. (2015). Pulmonary rehabilitation for chronic obstructive pulmonary disease. Cochrane Database Syst. Rev..

[5] Puhan M.A., Gimeno-Santos E., Cates C.J., Troosters T. (2016). Pulmonary rehabilitation following exacerbations of chronic obstructive pulmonary disease. Cochrane Database Syst. Rev..

[6] Dowman L., Hill C.J., May A., Holland A.E. (2021). Pulmonary rehabilitation for interstitial lung disease. Cochrane Database Syst. Rev..

[7] Burnett K., Glaspole I., Holland A.E. (2019). Understanding the patient’s experience of care in idiopathic pulmonary fibrosis. Respirology.

[8] Lee A.L., Hill C.J., McDonald C.F., Holland A.E. (2017). Pulmonary Rehabilitation in Individuals With Non–Cystic Fibrosis Bronchiectasis: A Systematic Review. Arch. Phys. Med. Rehabil..

[9] Morris N.R., Kermeen F.D., Holland A.E. (2017). Exercise-based rehabilitation programmes for pulmonary hypertension. Cochrane Database Syst. Rev..

[10] Higashimoto Y., Ando M., Sano A., Saeki S., Nishikawa Y., Fukuda K., Tohda Y. (2020). Effect of pulmonary rehabilitation programs including lower limb endurance training on dyspnea in stable COPD: A systematic review and meta-analysis. Respir. Investig..

[11] Rochester C.L., Vogiatzis I., Holland A.E., Lareau S.C., Marciniuk D.D., Puhan M.A., Spruit M.A., Masefield S., Casaburi R., Clini E.M. (2015). An Official American Thoracic Society/European Respiratory Society Policy Statement: Enhancing Implementation, Use, and Delivery of Pulmonary Rehabilitation. Am. J. Respir. Crit..

[12] Cox N.S., Oliveira C.C., Lahham A., Holland A.E. (2017). Pulmonary rehabilitation referral and participation are commonly influenced by environment, knowledge, and beliefs about consequences: A systematic review using the Theoretical Domains Framework. J. Physiother..

[13] Roberts C.M., Gungor G., Parker M., Craig J., Mountford J. (2015). Impact of a patient-specific co-designed COPD care scorecard on COPD care quality: A quasi-experimental study. NPJ Prim. Care Respir. Med..

[14] Deprez R., Kinner A., Millard P., Baggott L., Mellett J., Loo J.L. (2009). Improving quality of care for patients with chronic obstructive pulmonary disease. Popul. Health Manag..

[15] Lange P., Rasmussen F.V., Borgeskov H., Dollerup J., Jensen M.S., Roslind K., Nielsen L.M. (2007). The quality of COPD care in general practice in Denmark: The KVASIMODO study. Prim. Care Respir. J..

[16] Tøttenborg S.S., Thomsen R.W., Nielsen H., Johnsen S.P., Hansen E.F., Lange P. (2013). Improving quality of care among COPD outpatients in Denmark 2008–2011. Clin. Respir. J..

[17] Harris M., Smith B.J., Veale A.J., Esterman A., Frith P.A., Selim P. (2009). Providing reviews of evidence to COPD patients: Controlled prospective 12-month trial. Chronic Respir. Dis..

[18] Zwar N.A., Hermiz O., Comino E., Middleton S., Vagholkar S., Xuan W., Wilson S.F., Marks G.B. (2012). Care of patients with a diagnosis of chronic obstructive pulmonary disease: A cluster randomised controlled trial. Med. J. Aust..

[19] Cox N.S., Dal Corso S., Hansen H., McDonald C.F., Hill C.J., Zanaboni P., Alison J.A., O’Halloran P., Macdonald H., Holland A.E. (2021). Telerehabilitation for chronic respiratory disease. Cochrane Database Syst. Rev..

[20] Milner S.C., Boruff J.T., Beaurepaire C., Ahmed S., Janaudis-Ferreira T. (2018). Rate of, and barriers and enablers to, pulmonary rehabilitation referral in COPD: A systematic scoping review. Respir. Med..

[21] Stone P.W., Hickman K., Steiner M.C., Roberts C.M., Quint J.K., Singh S.J. (2020). Predictors of Referral to Pulmonary Rehabilitation from UK Primary Care. Int. J. Chronic Obstr. Pulm. Dis..

[22] Katagira W., Jones A.V., Orme M.W., Yusuf Z.K., Ndagire P., Nanyonga J., Kasiita R., Kasolo J.N., Miah R.B., Steiner M.C. (2021). Identifying Appropriate Delivery of and Referral to Pulmonary Rehabilitation in Uganda: A Survey Study of People Living with Chronic Respiratory Disease and Health Care Workers. Int. J. Chronic Obstr. Pulm. Dis..

[23] Jones S.E., Green S.A., Clark A.L., Dickson M.J., Nolan A.-M., Moloney C., Kon S.S.C., Kamal F., Godden J., Howe C. (2014). Pulmonary rehabilitation following hospitalisation for acute exacerbation of COPD: Referrals, uptake and adherence. Thorax.

[24] Osadnik C., Gordon C., Gerstman E. (2019). Referrals to pulmonary rehabilitation after acute exacerbations of COPD: A mixed-methods evaluation. Eur. Respir. J..

[25] De Andrade J.A., Kulkarni T., Neely M.L., Hellkamp A.S., Case A.H., Guntupalli K., Bender S., Conoscenti C.S., Snyder L.D. (2021). Implementation of guideline recommendations and outcomes in patients with idiopathic pulmonary fibrosis: Data from the IPF-PRO Registry. Respir. Med..

[26] Rochester C.L., Spruit M.A., Holland A.E. (2021). Pulmonary Rehabilitation in 2021. JAMA.

[27] Desveaux L., Janaudis-Ferreira T., Goldstein R., Brooks D. (2015). An International Comparison of Pulmonary Rehabilitation: A Systematic Review. J. Chronic Obstr. Pulm. Dis..

[28] Moscovice I.S., Casey M.M., Wu Z. (2019). Disparities in Geographic Access to Hospital Outpatient Pulmonary Rehabilitation Programs in the United States. Chest.

[29] Johnston C.L., Maxwell L.J., Alison J.A. (2011). Pulmonary rehabilitation in Australia: A national survey. Physiotherapy.

[30] Camp P.G., Hernandez P., Bourbeau J., Kirkham A., Debigare R., Stickland M.K., Goodridge D., Marciniuk D.D., Road J.D., Bhutani M. (2015). Pulmonary rehabilitation in Canada: A report from the Canadian Thoracic Society COPD Clinical Assembly. Can. Respir. J..

[31] Perez X., Wisnivesky J.P., Lurslurchachai L., Kleinman L.C., Kronish I.M. (2012). Barriers to adherence to COPD guidelines among primary care providers. Respir. Med..

[32] Glaab T., Vogelmeier C., Hellmann A., Buhl R. (2012). Guideline-based survey of outpatient COPD management by pulmonary specialists in Germany. Int. J. Chronic Obstr. Pulm. Dis..

[33] Perera C., Jayamaha A.R., Orme M.W., Jones A.V., Amarasekara A.A.T.D., Barton A., Steiner M.C., Jones R., Wimalasekera S.W., Singh S.J. (2020). Opinion and practice of pulmonary rehabilitation amongst health care providers in selected areas of Sri Lanka. Eur. Respir. J..

[34] Dummer J., Tumilty E., Hannah D., McAuley K., Baxter J., Doolan-Noble F., Donlevy S., Stokes T. (2020). Health Care Utilisation and Health Needs of People with Severe COPD in the Southern Region of New Zealand: A Retrospective Case Note Review. J. Chronic Obstr. Pulm. Dis..

[35] Steiner M., Holzhauer-Barrie J., Lowe D., Searle L., Skipper E., Welham S., Roberts C. (2015). Pulmonary Rehabilitation: Time to Breathe Better. National Chronic Obstructive Pulmonary Disease (COPD) Audit Programme: Resources and Organisation of Pulmonary Rehabilitation Services in England and Wales 2015. National Organisational Audit Report.

[36] McNaughton A.A., Weatherall M., Williams G., Delacey D., George C., Beasley R. (2016). An audit of pulmonary rehabilitation program. Clin. Audit.

[37] O’Shea S.D., Taylor N.F., Paratz J.D. (2007). But watch out for the weather: Factors affecting adherence to progressive resistance exercise for persons with COPD. J. Cardiopulm Rehabil. Prev..

[38] Fischer M.J., Scharloo M., Abbink J.J., Thijs-Van A., Rudolphus A., Snoei L., Weinman J.A., Kaptein A.A. (2007). Participation and drop-out in pulmonary rehabilitation: A qualitative analysis of the patient’s perspective. Clin. Rehabil..

[39] British Lung Foundation Chronic Obstructive Pulmonary Disease (COPD) Statistics. https://www.blf.org.uk/.

[40] Keating A., Lee A., Holland A.E. (2011). What prevents people with chronic obstructive pulmonary disease from attending pulmonary rehabilitation? A systematic review. Chronic Respir. Dis..

[41] Moore L., Hogg L., White P. (2012). Acceptability and feasibility of pulmonary rehabilitation for COPD: A community qualitative study. Prim. Care Respir. J..

[42] Papps E., Ramsden I. (1996). Cultural safety in nursing: The New Zealand experience. Int. J. Qual. Health Care.

[43] Levack W.M., Jones B., Grainger R., Boland P., Brown M., Ingham T.R. (2016). Whakawhanaungatanga: The importance of culturally meaningful connections to improve uptake of pulmonary rehabilitation by Māori with COPD—A qualitative study. Int. J. Chronic Obstr. Pulm. Dis..

[44] Arnold E., Bruton A., Ellis-Hill C. (2006). Adherence to pulmonary rehabilitation: A qualitative study. Respir. Med..

[45] Harrison S.L., Robertson N., Apps L., Steiner M.C., Morgan M.D.L., Singh S.J. (2015). “We are not worthy”—Understanding why patients decline pulmonary rehabilitation following an acute exacerbation of COPD. Disabil. Rehabil..

[46] Bulley C., Donaghy M., Howden S., Salisbury L., Whiteford S., Mackay E. (2009). A prospective qualitative exploration of views about attending pulmonary rehabilitation. Physiother. Res. Int..

[47] Johnston K., Young M., Grimmer K., Antic R., Frith P. (2013). Frequency of referral to and attendance at a pulmonary rehabilitation programme amongst patients admitted to a tertiary hospital with chronic obstructive pulmonary disease. Respirology.

[48] Keating A., Lee A.L., Holland A.E. (2011). Lack of perceived benefit and inadequate transport influence uptake and completion of pulmonary rehabilitation in people with chronic obstructive pulmonary disease: A qualitative study. J. Physiother..

[49] Hayton C., Clark A., Olive S., Browne P., Galey P., Knights E., Staunton L., Jones A., Coombes E., Wilson A.M. (2013). Barriers to pulmonary rehabilitation: Characteristics that predict patient attendance and adherence. Respir. Med..

[50] Ansari Z., Dunt D., Dharmage S.C. (2007). Variations in hospitalizations for chronic obstructive pulmonary disease in rural and urban Victoria, Australia. Respirology.

[51] Doyle D., Tommarello C., Broce M., Emmett M., Pollard C. (2017). Implementation and outcomes of a community-based pulmonary rehabilitation program in rural Appalachia. J. Cardiopulm Rehabil. Prev..

[52] Johnston C.L., Maxwell L.J., Maguire G.P., Alison J.A. (2014). Does delivery of a training program for healthcare professionals increase access to pulmonary rehabilitation and improve outcomes for people with chronic lung disease in rural and remote Australia?. Aust. Health Rev..

[53] Brooke M.E., Spiliopoulos N., Collins M. (2017). A review of the availability and cost effectiveness of chronic obstructive pulmonary disease (COPD) management interventions in rural Australia and New Zealand. Rural Remote Health.

[54] Farah R., Groot W., Pavlova M. (2020). Barriers to access to pulmonary rehabilitation in developing countries: A systematic review. Virtual Congress 2020—Optimising the benefits of pulmonary rehabilitation. Eur. Respir. J..

[55] Sami R., Salehi K., Hashemi M., Atashi V. (2021). Exploring the barriers to pulmonary rehabilitation for patients with chronic obstructive pulmonary disease: A qualitative study. BMC Health Serv. Res..

[56] Habib G.M.M., Rabinovich R., Divgi K., Ahmed S., Saha S.K., Singh S., Uddin A., Uzzaman M.N., Pinnock H. (2020). Systematic review of clinical effectiveness, components, and delivery of pulmonary rehabilitation in low-resource settings. NPJ Prim. Care Respir. Med..

[57] Orme M.W., Free R.C., Manise A., Jones A.V., Akylbekov A., Barton A., Emilov B., Girase B., Jayamaha A.R., Jones R. (2020). Global RECHARGE: Establishing a standard international data set for pulmonary rehabilitation in low- and middle-income countries. J. Glob. Health.

[58] Katagira W., Orme M.W., Jones A.V., Kasiita R., Jones R., Barton A., Miah R.B., Manise A., Matheson J.A., Free R.C. (2021). Study protocol for a randomised controlled trial assessing the impact of pulmonary rehabilitation on maximal exercise capacity for adults living with post-TB lung disease: Global RECHARGE Uganda. BMJ Open.

[59] Jayamaha A.R., Perera C.H., Orme M.W., Jones A.V., Wijayasiri U.K.D.C., Amarasekara T.D., Karunatillake R.S., Fernando A., Seneviratne A.L.P., Barton A. (2020). Protocol for the cultural adaptation of pulmonary rehabilitation and subsequent testing in a randomised controlled feasibility trial for adults with chronic obstructive pulmonary disease in Sri Lanka. BMJ Open.

[60] Harris D., Hayter M., Allender S. (2008). Improving the uptake of pulmonary rehabilitation in patients with COPD: Qualitative study of experiences and attitudes. Br. J. Gen. Pract..

[61] Early F., Wellwood I., Kuhn I., Deaton C., Fuld J. (2018). Interventions to increase referral and uptake to pulmonary rehabilitation in people with COPD: A systematic review. Int. J. Chronic Obstr. Pulm. Dis..

[62] Kaufmann C., Markun S., Hasler S., Lana K.D., Rosemann T., Senn O., Steurer-Stey C. (2015). Performance Measures in the Management of Chronic Obstructive Pulmonary Disease in Primary Care—A Retrospective Analysis.

[63] Canadian Pulmonary Rehabilitation Program. https://www.livingwellwithcopd.com/canadian-pulmonary-rehabilitation-program.html.

[64] Jones A.W., Taylor A., Gowler H., O’Kelly N., Ghosh S., Bridle C. (2017). Systematic review of interventions to improve patient uptake and completion of pulmonary rehabilitation in COPD. ERJ Open Res..

[65] Ringbaek T.J., Lavesen M., Lange P. (2016). Tablet computers to support outpatient pulmonary rehabilitation in patients with COPD. Eur. Clin. Respir. J..

[66] WHO Global Health Estimates. https://www.who.int/news-room/fact-sheets/detail/chronic-obstructive-pulmonary-disease-.

[67] Fortney J.C., Pyne J.M., Edlund M.J., Williams D.K., Robinson D.E., Mittal D., Henderson K.L. (2007). A randomized trial of telemedicine-based collaborative care for depression. J. Gen. Intern. Med..

[68] Charles B.L. (2000). Telemedicine can lower costs and improve access. Healthc. Financ. Manag..

[69] Kruse C.S., Krowski N., Rodriguez B., Tran L., Vela J., Brooks M. (2017). Telehealth and patient satisfaction: A systematic review and narrative analysis. BMJ Open.

[70] Osadnik C.R. (2018). Moving pulmonary rehabilitation forwar.rd in COPD: Stepping towards (home-based) action. Respirology.

[71] Hansen H., Bieler T., Beyer N., Kallemose T., Wilcke J.T., Østergaard L.M., Frost Andeassen H., Martinez G., Lavesen M., Frølich A. (2020). Supervised pulmonary tele-rehabilitation versus pulmonary rehabilitation in severe COPD: A randomised multicentre trial. Thorax.

[72] Holland A.E., Mahal A., Hill C.J., Lee A.L., Burge A.T., Cox N.S., Moore R., Nicolson C., O’Halloran P., Lahham A. (2017). Home-based rehabilitation for COPD using minimal resources: A randomised, controlled equivalence trial. Thorax.

[73] Horton E.J., Mitchell K.E., Johnson-Warrington V., Apps L.D., Sewell L., Morgan M., Taylor R.S., Singh S.J. (2018). Comparison of a structured home-based rehabilitation programme with conventional supervised pulmonary rehabilitation: A randomised non-inferiority trial. Thorax.

[74] Maltais F., Bourbeau J., Shapiro S., Lacasse Y., Perrault H., Baltzan M., Hernandez P., Rouleau M., Julien M., Parenteau S. (2008). Effects of home-based pulmonary rehabilitation in patients with chronic obstructive pulmonary disease: A randomized trial. Ann. Intern. Med..

[75] Neves L.F., Reis M.H.D., Gonçalves T.R. (2016). Home or community-based pulmonary rehabilitation for individuals with chronic obstructive pulmonary disease: A systematic review and meta-analysis. Cad. Saúde Pública.

[76] Wuytack F., Devane D., Stovold E., McDonnell M., Casey M., McDonnell T.J., Gillespie P., Raymakers A., Lacasse Y., McCarthy B. (2018). Comparison of outpatient and home-based exercise training programmes for COPD: A systematic review and meta-analysis. Respirology.

[77] Bondarenko J., Babic C., Burge A.T., Holland A.E. (2020). Home-based pulmonary rehabilitation: An implementation study using the RE-AIM framework. ERJ Open Res..

[78] Wiemers E.E., Abrahams S., AlFakhri M., Hotz V.J., Schoeni R.F., Seltzer J.A. (2020). Disparities in Vulnerability to Severe Complications from COVID-19 in the United States. MedRxiv.

[79] Spinelli A., Pellino G. (2020). COVID-19 pandemic: Perspectives on an unfolding crisis. J. Br. Surg..

[80] Rochester C.L., Langer D., Singh S.J., Holland A.E., Dal Corso S., Spruit M.A. (2021). What does the future hold for pulmonary rehabilitation?. ERS Monograph.

[81] Monaghesh E., Hajizadeh A. (2020). The role of telehealth during COVID-19 outbreak: A systematic review based on current evidence. BMC Public Health.

[82] Dechman G., Aceron R., Beauchamp M., Bhutani M., Bourbeau J., Brooks D., Goldstein R., Goodridge D., Hernandez P., Janaudis-Ferreira T. (2020). Delivering pulmonary rehabilitation during the COVID-19 pandemic: A Canadian Thoracic Society position statement. Can. J. Respir. Crit. Care Sleep Med..

[83] Tsutsui M., Gerayeli F., Sin D.D. (2021). Pulmonary Rehabilitation in a Post-COVID-19 World: Telerehabilitation as a New Standard in Patients with COPD. Int. J. Chronic Obstr. Pulm. Dis..

